# A comprehensive review of the supraspinal mechanisms of spinal cord stimulation on chronic pain and cognition

**DOI:** 10.3389/fpain.2025.1589723

**Published:** 2025-07-28

**Authors:** Kelly E. Gartner, Sofia Rustioni, Aamya Vohra, Mustafa Almosawi, Norah Hill, Travis Stewart, Nelleke C. van Wouwe, Ajmal Zemmar

**Affiliations:** ^1^ Department of Neurological Surgery, University of Louisville School of Medicine, Louisville, KY, United States; ^2^ University of Louisville, Louisville, KY, United States; ^3^ University of Louisville School of Medicine, Louisville, KY, United States; ^4^ Kentucky Spinal Cord Injury Research Center, University of Louisville, Louisville, KY, United States; ^5^ Department of Neurosurgery, Robley Rex VA Medical Center, Louisville, KY, United States

**Keywords:** SCS, chronic pain, drug-refractory pain, supraspinal effects, neuroanatomy, cognitive function

## Abstract

Chronic pain is one of the leading causes of disability worldwide. It can result in a significant reduction in quality of life and has been associated with decreased neurocognitive performance in attention, memory, and processing speed. Spinal cord stimulation (SCS) is a surgical treatment option for drug-refractory chronic pain. Although SCS can improve pain perception and related physical well-being, the mechanisms by which SCS improves pain perception and affects cognition remain largely unknown. Here, we review the cognitive impairments and neuroanatomical changes that can arise from chronic pain and how SCS treatment impacts these. This review identifies four key regions that may modulate attention, executive and emotional functioning, and memory with SCS: the amygdala, anterior cingulate cortex, thalamus, and somatosensory cortex. These observations suggest a role for SCS to influence and modulate the cognitive-emotional aspects of pain perception. Our review provides new insights to identify potential cortical areas that can serve as biomarkers or neuromodulation targets for SCS treatment. Recognizing the changes in activity within these supraspinal regions during SCS treatment may help individualize pain treatment and induce favorable cognitive shifts.

## Introduction

Chronic pain is one of the leading causes of disability worldwide (1, 2). Chronic pain evolves from pain lasting longer than three months and can arise from inflammation, neuropathy, or idiopathy (2). This long-term pain can result in a significant reduction in quality of life and has been associated with decreased neurocognitive performance in attention, memory, and executive functioning (3). Spinal cord stimulation (SCS) is a surgical treatment option for drug-refractory chronic pain (4). Although SCS can improve pain perception and related physical well-being, the impact of SCS on cognition in patients with chronic pain is not fully understood. The purpose of this review is to evaluate the impact of SCS treatment for chronic pain on the supraspinal areas important for cognitive function. This review discusses the supraspinal changes and cognitive impairments that can arise from chronic pain, as well as the changes associated with SCS treatment, in order to identify overlapping biomarkers. We suggest that SCS-induced activity changes within the amygdala, anterior cingulate cortex, thalamus, and somatosensory cortex (5, 6) may modulate various cognitive processes, including attention, executive and emotional processing, and memory. This comprehensive review serves to emphasize the neural intricacies of chronic pain, elucidate the supraspinal effects of SCS, and further inform SCS parameters to treat chronic pain while minimizing cognitive consequences.

## Supraspinal structures processing pain

Acute pain originates from receptors in the skin or tissue that transduce chemical, mechanical, and thermal pain into nociceptive signals. These signals are relayed via peripheral primary afferent nociceptors to the dorsal horn of the spinal cord. There, the primary afferents synapse with secondary afferents that transmit the signals to supraspinal cortical and subcortical regions (7). Nociceptive information is also relayed to the brainstem, midbrain, and medullary regions, which can modulate the perception and sensation of noxious stimuli (7–10). Pain perception results from the activation of an intricate network of connections within the cortex involving sensory, emotional, and cognitive processes (11); these pathways can be activated and cause painful sensations even in the absence of nociceptive input by direct stimulation (12).

Chronic pain alters both lateral and medial ascending spinothalamic and descending pain pathways (13–15). The lateral pathway processes the sensory aspects of pain and includes projections from the spinal cord towards the ventral posterolateral thalamus and subsequently to parietal and sensorimotor cortex (13, 15). The medial pathway processes the affective and motivational aspects of pain and involves spinal projections to the midline and ventral posterolateral nuclei of the thalamus and then connects to the dorsal anterior cingulate cortex and insula. The descending pain inhibitory pathway modulates pain perception by suppressing nociceptive input. Key structures in this network include pregenual anterior cingulate cortex, periaqueductal gray, and the ventromedial medulla (13, 15).

The main supraspinal regions involved in processing pain include the primary and secondary somatosensory cortices, primary and supplementary motor cortices, insular cortex, prefrontal cortex, parietal cortex, anterior cingulate cortex, thalamus, amygdala, hippocampus, and basal ganglia (12). The thalamus and anterior cingulate cortex play a role in registering stimuli as painful, while the somatosensory cortex distinguishes non-painful stimuli (16). One study showed that the primary somatosensory cortex and the insular cortex had increased activity associated with the intensity of the painful stimulus (12). These sensory cortical areas may additionally modulate context-dependent pain processes and the overall perception of pain (12). The motor and supplementary motor cortices are thought to process the motivational or escape aspects of experiencing pain (17), while the prefrontal cortex likely drives descending modulation of pain (12, 18). The prefrontal cortex plays a multifaceted role in pain processing through several of its subregions, including the medial prefrontal cortex, dorsolateral prefrontal cortex, orbitofrontal cortex, and ventrolateral prefrontal cortex (19). The medial prefrontal cortex and dorsolateral prefrontal cortex are involved in pain inhibition (through connections with periaqueductal gray and corticostriatial circuits), whereas orbitofrontal cortex and ventrolateral prefrontal cortex are important for regulating affective aspects of pain (19). Specifically, a low-frequency enhancement of basal activity in the prefrontal cortex linearly increases prefrontal outputs to inhibit pain, and a decrease in basal firing leads to a reduction in pain-evoked responses (18). This indicates a cortical gain control system where the prefrontal cortex regulates pain processing through top-down feedback. The brainstem, midbrain, and medullary areas also modulate descending pain signals in the spinal circuitry through both inhibition and excitation (12, 20, 21). The subcortical structures of the basal ganglia could play a role in discriminating pain intensity, coordinating motor responses, and influencing motivational aspects of pain (12). While processing acute pain involves a vast and intertwined circuitry, processing chronic pain may require even higher complexity as plasticity induces both connectivity and structural changes to the nociceptive network over time.

## Effects of chronic pain on supraspinal structures and connectivity

Chronic pain may prompt the reorganization of supraspinal activity, shifting the representation of pain gradually from sensory structures to emotional and limbic regions. Long-term potentiation, where the persistent activation of a neuronal connection increases its synaptic strength, leads to a more sensitive and efficacious response to stimuli (22). While there are benefits to long-term potentiation, like encoding and consolidating memories (22–24), neuronal sensitization within nociceptive pathways can cause pain hypersensitivity (25). Conversely, long-term depression, the persistent reduction of synaptic strength, can inactivate memory (22) and reduce pain sensitivity (26). These plastic changes may persist in a memory-like fashion even after the original source of the pain is removed or cured (27). Chronic pain may result due to persistence of this synaptic strengthening in memory-like fashion. Phantom limb sensations, for example, result from somatosensory inputs of pain of a significant intensity and duration that can produce lasting changes and sensitivities within the central nervous system (28). Single-occurrence nociceptive signals mediated by the limbic circuitry extinguish over time, but the progression from acute to chronic pain instigates activity-induced plasticity within the limbic-cortical circuitry (29, 30). Patients with chronic pain exhibit decreased volume and biochemical plasticity of the hippocampus (31, 32) and hyperactivity within the amygdala (30). These pathways can shift the single-occurrence nociceptive state to emotional learning by increasing activity in prefrontal cortical circuits (29, 33). Specifically, hyperactivity of the amygdala during pain generates feedforward inhibition, thereby deactivating the medial prefrontal cortex. This subsequently impairs top-down cortical control mechanisms (30) and couples activity between the thalamus and medial prefrontal cortex (16). A functional magnetic resonance imaging (fMRI) study found decreased resting state functional activity and synchrony between the thalamus and primary somatosensory cortex in patients with peripheral neuropathic pain when compared to healthy, age-matched, and gender-matched controls (34). Furthermore, the ventral posterior lateral nucleus of the thalamus had reduced synchrony with sensorimotor, frontal, cingulate, and parietal areas, while the medial dorsal thalamic nucleus exhibited reduced synchrony with more sensory and emotional processing areas (34). Together, these studies support the theory that chronic pain alters limbic-cortical and thalamocortical connections, shifting pain processing away from sensory structures.

Many electroencephalogram (EEG) studies have observed slowing of the dominant alpha peak in pain patients with fibromyalgia (35), chronic pancreatitis (36), neurogenic pain (37), and capsaicin-heat pain (38), indicating that lower peak alpha frequency may be a biomarker for pain. Additionally, compared to healthy controls, patients with fibromyalgia showed increases in theta, beta, and gamma power (35). Beta and gamma over-activation was observed in the prefrontal cortex, orbitofrontal cortex, insular cortex, primary motor cortex, and primary and secondary somatosensory cortices, while increased theta was mostly localized to the prefrontal cortex and orbitofrontal cortex (35). Spectral power was also higher over the frequency range of 2–25 Hz in non-medicated patients with neurogenic pain compared to healthy controls (37). A study in rats proposed that chronic pain decreases spontaneous and pain-evoked basal firing rates in the prefrontal cortex, and that low-frequency optogenetic stimulation within the prefrontal cortex could provide the cortical gain control necessary to effectively treat pain (18). However, deep brain and motor cortex stimulation are not currently approved for chronic pain in humans (39).

In addition to shifts in activity and functional connectivity, there may also be larger-scale structural changes in the chronic pain population. Patients with chronic migraine had significantly reduced cortical thickness in the insular cortex, caudal middle frontal gyrus, precentral cortex, and parietal cortex compared to healthy controls (40). The number of days patients had a migraine during the month preceding the MRI was negatively correlated with the thickness of the right insular cortex (i.e., as days of migraine increased, cortical thickness decreased) (40). There is also a significant correlation between duration of episodic migraine history in years and gray matter volume in the right pars opercularis, right superior frontal gyrus, and left insular cortex (41). Another study measured gray and white matter changes in younger (29–49 years old) and older (51–60 years old) patients with fibromyalgia and compared them to age-matched and medication-matched controls (42). Older patients showed decreased gray matter and loss of white matter integrity, but younger patients showed gray matter hypertrophy and associated white matter changes. Gray matter decreases in older patients compared to age-matched controls were located in the anterior cingulate cortex, posterior cingulate cortex, prefrontal cortical areas, and premotor cortex. Gray matter hypertrophy in younger patients compared to age-matched controls was located in the insular cortex, basal ganglia, and prefrontal cortex. In both groups, anatomical changes were associated with the patient's sensitivity to pain—an increased experimental pain sensitivity in older patients, and normal pain sensitivity in younger patients (42).

These studies support the idea that pain processing can alter neuroanatomy and connectivity, and emphasize pain duration as a key factor contributing to the structural plasticity of pain patients. Although the anatomical changes seen in younger individuals with fibromyalgia may seem adaptive, the increases in gray matter may be caused by the over-engagement of pain modulatory networks and potentially attenuate the perception of painful stimuli (42). However, it is uncertain whether these anatomical changes are associated with observed electrophysiological changes in patients with chronic pain, including lower peak alpha frequency, increased spectral power, increased theta, beta, and gamma power, and the desynchrony of the thalamus and sensory cortical areas. Future studies elucidating the relationship between these neuroanatomical and functional changes could aid SCS settings and location targeting for chronic pain treatment and help determine the effects of SCS on other cognitive networks.

In sum:

• Chronic pain leads to changes in supraspinal neuroanatomy and functional activity across the lateral and medial ascending spinothalamic and descending pain pathways.
• Chronic pain alters limbic-cortical and thalamocortical connectivity and anatomy, causing a shift in brain activity from sensory regions to emotional and limbic structures.

## Effects of chronic pain on cognition

Long-term chronic pain can cause neuroanatomical and connectivity changes that may directly influence cognitive processes, including attention, executive functioning, and memory (6, 29, 33, 40–53). Chronic pain research has the inherent limitation that patients may be on opioid medication or other analgesics that could influence cognition (54–57). However, other studies report that opioid medication ameliorates or has no effect on cognitive performance when compared with unmedicated patients (58–60). Analgesics are difficult to control in chronic pain patients due to ethical reasons, making medication and the addiction to medication potential confounding factors in studies investigating the cognitive effects of chronic pain.

Patients suffering from high-intensity chronic pain have significant attentional deficits compared to healthy controls (61). However, this may be severity-specific. Studies have shown attention is only impaired in difficult tasks in patients with high pain levels (62, 63) and that patients with low-intensity chronic pain do not perform differently than controls on an executive control task [adapted color-word Stroop task (63)]. One study measured attention performance (Test of Everyday Attention) alongside pain severity and interference testing (Brief Pain Inventory) in 354 pain patients (64). This study found that pain interference scores were inversely associated with selective attention, such that when pain interference was high, selective attention was low. Pain severity was also associated with poorer selective and sustained attention (64). Overlaps between the attentional network and the pain network have been found, and it has been theorized that the competition between pain and normal functioning in these centers may result in decreased attentional control and task performance (46). Specifically, the insular and midcingulate cortex control bottom-up attention to nociceptive signals, whereas prioritizing a goal or task is supported by the prefrontal cortex (46). Distraction from pain by a task that requires attentional resources, like the Stroop task, decreased self-reported pain intensity and unpleasantness in young and middle-aged adults (43, 52). Distraction from pain is accompanied by a reduced activation within areas of the pain network, including the insular cortex, thalamus, prefrontal cortex, midcingulate cortex, and the cognitive division of the anterior cingulate cortex (43, 52). However, the affective division of the anterior cingulate cortex, as well as the orbitofrontal cortex, showed increased activity (43). In contrast, when attention is directed at a painful stimulus, the amygdala (30), prefrontal cortical areas, and the thalamus are activated (65), inducing plasticity between the limbic-cortical circuitry and within the pain pathway (16). Together, these studies highlight the overlap of pain and attentional networks and help elucidate the attentional impairments associated with pain.

Executive functioning includes a number of higher-order cognitive processes that control, organize, and direct actions, emotional responses, and behaviors (66). One theory proposes that more complex neurological processes required for executive control tasks—such as working memory, planning, organization, control of conflicting thoughts, goal-directed behavior, assessing the consequences of actions, and emotional decision-making—are more likely to be impaired by chronic pain than less complex automatic processes like implicit memory (31, 67, 68). Furthermore, the cognitive effects of chronic pain may depend on pain intensity and type (31). A study compared emotional decision-making under uncertainty (IOWA gambling task) of patients with chronic back pain, chronic complex regional pain syndrome, and healthy controls (69). Both chronic pain groups had significantly poorer decision-making performance than controls, indicating difficulty with decision-making under uncertainty or learning from feedback. Moreover, only the pain intensity of the chronic back pain group correlated with decision-making performance (69). Interestingly, there is a correlation between the thickness of the right insular cortex and the severity of clinical pain (migraine) (40) that may influence processes like attention (44). Additionally, executive function is regulated by the anterior cingulate cortex (70) and may be impacted by pain-induced changes in activity (42). Indeed, decreased volume or altered activity in the anterior cingulate cortex can reduce processing speed, alter cognitive executive functioning, and lead to emotional impairments (6, 40, 41, 50). Emotional processing may also be affected by the implication of the amygdala in the pain network (6) and the hyperactivity of the amygdala during pain (16, 30). The amygdala is important for emotional processing and effectively avoiding harmful stimuli (71, 72). However, persistently increased amygdala activity during pain could lead to plasticity changes within the thalamus and the medial prefrontal cortex (16). Overall, executive functions such as emotional processing, processing speed, and effective decision-making can potentially be altered in patients with chronic pain due to the involvement of the insular cortex, anterior cingulate cortex, and the amygdala in the pain pathway.

Patients with chronic pain often complain of difficulties with memory and have reduced working memory, recognition memory, and short-term recall (51, 73–76). However, literature suggests implicit memory is less likely to be affected by chronic pain (63). There are many mechanistic correlates relating chronic pain and memory [for review see (33)]. As the pain system demonstrates higher-order plasticity via both long-term depression and long-term potentiation (26), the progression from acute to chronic pain can instigate activity-induced plasticity within the limbic-cortical circuitry (30). The limbic regions of the amygdala and hippocampus are integral to learning and memory functions (33, 45, 47–49, 53). Deficits in working memory were associated with decreased dendritic complexity within the hippocampus of mice with chronic pain (51). Patients with chronic pain have decreased levels of brain-derived neurotrophic factors (BDNF) in the hippocampus, indicating less plasticity—a finding that is linked to depression-like symptoms (77, 78). Chronic pain is often associated with comorbid affective disorders, such as depression, anxiety, and sleep disorders, that may impair the results of cognitive and functional testing (75). Chronic pain patients, particularly those with comorbid anxiety and depression, suffer significant decreases in working memory, recall, and recognition memory (75). Specifically, intercorrelations were found between memory and anxiety (*r* = 0.53, *P* < 0.001) and memory and depression (*r* = 0.60, *P* < 0.001) in 149 patients with benign chronic pain (75). This relationship may be partially due to the overlapping roles of the amygdala in pain processing, memory, and depression and anxiety disorders. Therefore, when assessing the cognitive function of patients with chronic pain, comorbidities must be considered as they can exacerbate symptoms or lead to additional complications.

In sum:

• Chronic pain is associated with attention deficits, impaired executive function, reduced working memory, and recognition memory.
• This is likely due to altered activity in supraspinal regions across the pain pathways that are also involved in these cognitive functions, including the insula, cingulate cortex, prefrontal cortex, amygdala, and hippocampus.

## Effects of SCS on supraspinal structures and connectivity

**Figure 1:**
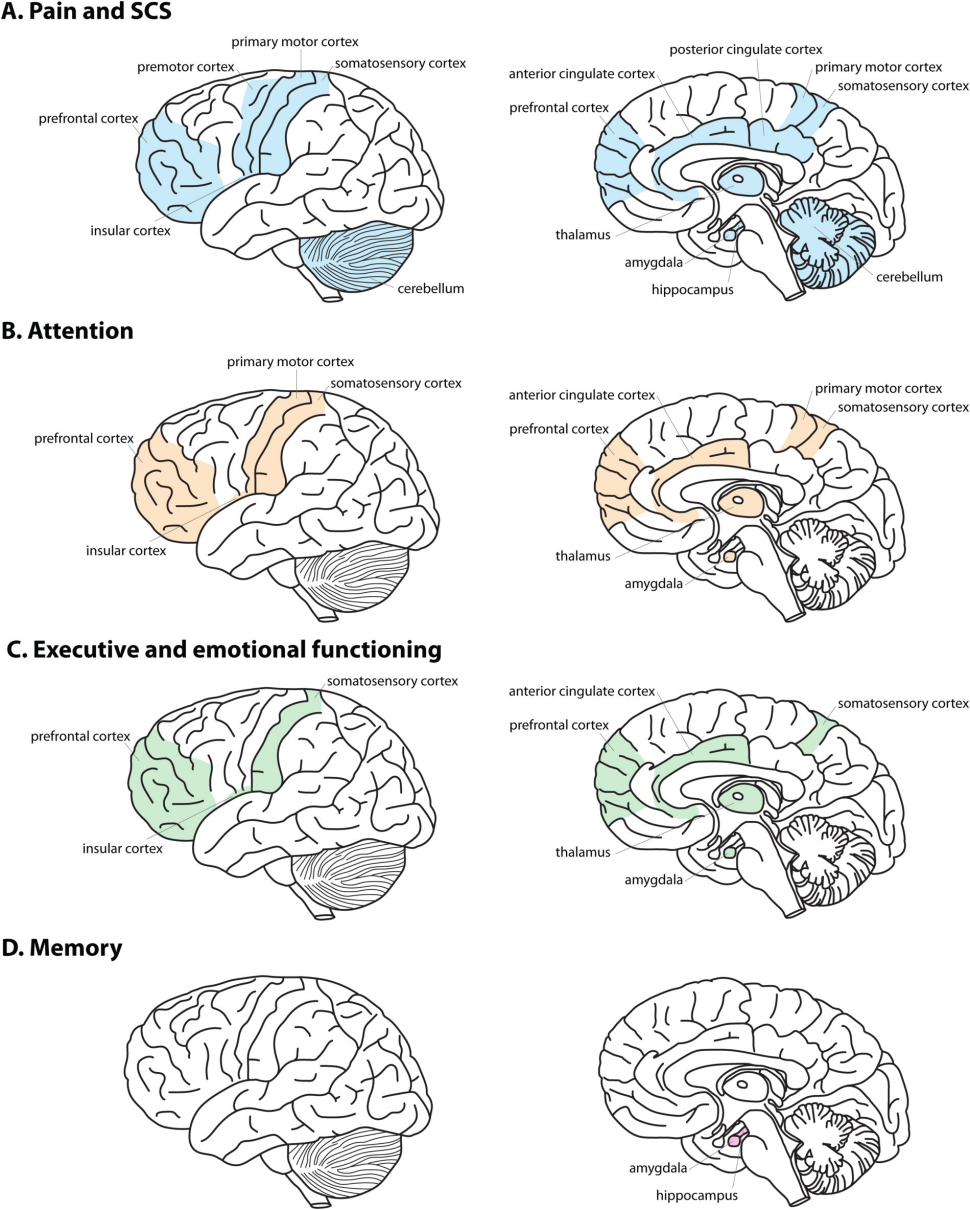
Supraspinal regions with overlap of pain, SCS-induced changes and cognitive effects. **(A)** Overlapping regions of pain networks and SCS activation (blue) may affect **(B)** attention (orange), **(C)** executive and emotional functioning (green), and **(D)** memory (pink).

SCS is a minimally invasive treatment option for drug-refractory chronic pain, with positive influences on pain perception and related physical well-being. The traditional implantable SCS system includes the SCS leads, extension wires, and an implantable pulse generator. The complications of implanted SCS systems may include electrode migration (13%–22%) (79), tolerance of SCS after one year (10%–29%) (79), and infection (∼5%) (80). However, the effects of SCS on pain and cognition may vary depending on the delivery method (i.e., paddle leads, percutaneous leads, or transcutaneous SCS), stimulation settings (i.e., burst, tonic, high frequency, low frequency), and location of stimulation on the spine. The stimulation settings and location can further vary based on the purpose of treatment [i.e., spinal cord injury (81), Parkinson's disease (82), gait/posture (83), chronic pain] and the condition of the patient. Burst stimulation consists of closely packed high-frequency pulses (five pulses at 500 Hz) followed by a quiescent period (13, 84, 85). In contrast, tonic stimulation uses a consistent stream of pulses at a lower frequency (30–70 Hz) (13, 85). An additional consideration is that conventional tonic stimulation (30–100 Hz) may cause paresthesia, while burst stimulation, high-dose tonic stimulation (500 Hz), and high frequency stimulation (10 kHz) are relatively paresthesia-free (86). These paresthesia-free, burst SCS paradigms are suggested to modulate the lateral, medial and descending pain pathways, while conventional tonic stimulation primarily modulates the lateral and descending pain pathways (86). The field has identified specific differences in regional brain activation between tonic and burst stimulation (13, 87). Additionally, studies have shown that burst SCS, compared to tonic SCS, has a greater clinical effectivity of pain reduction, and potentially aids the emotional and cognitive aspects of pain (82, 88, 89). De Ridder and Vanneste postulate that superior results from burst stimulation may be due to the additional modulation exerted on the medial pain pathway, the dorsal anterior cingulate cortex projections to the insular cortex, providing direct modulation of the spinothalamic pathways (13). Indeed, greater modulation of metabolic activity in the dorsal anterior cingulate cortex and posterior cingulate cortex was observed during burst SCS compared to tonic SCS (90). Furthermore, these regional metabolic rates correlated with PVAQ and VAS pain scales (90). Vetkas and colleagues support the argument that SCS frequency parameters differently influence brain activation by showing that low-frequency SCS (40 Hz) increased blood oxygen level dependent (BOLD) fMRI signals in the prefrontal cortex, while high-frequency SCS (1,200 Hz) increased BOLD activity in the somatosensory cortices, supplementary motor area, right insula, right posterior cingulate cortex, and orbitofrontal cortex (91), providing more extensive brain activation. EEG revealed an overall greater power spectrum and increased flow of information with high-dose SCS, with an increased frequency and pulse width and reduced amplitude, compared to conventional SCS (92). Overall, suprathreshold SCS, independent of frequency (4 Hz, 60 Hz, 500 Hz, 1 kHz), resulted in greater activity of frontal brain regions compared to subthreshold SCS (93). Additionally, evoked compound action potential (ECAP)-controlled closed-loop SCS may provide greater and more clinically significant pain relief compared to open-loop, fixed-output SCS systems (94). Future studies are necessary to directly compare the pain reduction and cognitive improvements between SCS settings and across neurological conditions, especially since studies with consistent medication protocols are limited due to ethical considerations (Table 1). The optimal SCS settings remain largely situationally dependent, and adjusting SCS parameters to optimize pain management with minimal cognitive ramifications is critical, as the broad effects of SCS on supraspinal structures span key areas within both cognitive and pain networks. Figure 1A shows the overlapping regions between pain networks and SCS activation networks.

**Table 1:** The effects of spinal cord stimulation on pain and supraspinal regions in medication-controlled studies.

Author and year	Diagnosis	SCS location	SCS protocol	Pain effects	Supraspinal effects
Deogaonkar et al. 2015 (100)	CRPS or neuropathic leg pain	Top of lead at T6–T9	Type not specified, duration >3 months Halted 2 h before imaging	Average 45% reduction in pain, with a range of 0%–75% Inclusion criteria were >50% pain reduction in >3 months with SCS	fMRI revealed decreased connectivity between somatosensory and limbic/emotional networks with SCS and pain relief Increased integration of somatosensory regions and the default mode network with SCS and pain relief
Kishima et al. 2010 (96)	Chronic neuropathic pain in lower limbs	Lumbar and Thoracic	6–12 months SCS SCS off for 12 h before study, pre-SCS scans, 30 min habitual bipolar stimulation, post-SCS scans	VAS pain levels were significantly reduced with an average decrease from 76.1 ± 25.2 before SCS to 40.6 ± 4.5 after SCS	PET revealed activation in regions associated with pain modulation and emotional regulation, such as anterior cingulate cortex and prefrontal regions, specifically the dorsolateral prefrontal cortex
Moens et al., 2012 (101)	FBSS	T8–T11	SCS optimized to individual 60 Hz, 210–μs, 1.7–3.3 V during imaging	During imaging mean pain reduction ratio was 55.47%	fMRI revealed short-term inverse correlations between pain relief and brain activity changes in brainstem, rostral anterior cingulate cortex, cerebellum, and dorsolateral prefrontal cortex Greater pain relief had more significant deactivation in ipsilateral antero-medial part of the thalamus
Pahapill et al. 2023 (122)	PSPS type 2	Not specified	Nonparesthesia producing waveforms ranging from 1 month to 2.4 years in duration SCS off during imaging	Pain levels based on NRS with stimulator off ranged from 3 to 7	rsfcMRI revealed striatum network (caudate nucleus, putamen, globus pallidus) indexes were significantly lower in patients with persistent spinal pain syndrome type 2 with SCS than in age-matched controls No correlation between reported pain levels and patient striatum network indexes, but striatum network of patients with constant neuropathic pain normalized and was directly correlated with duration of pain relief with SCS
Pahapill et al. 2024 (121)	PSPS type 2 with constant neuropathic vs. intermittent pain	Not specified	Nonparesthesia producing waveforms ranging from 6 month to 5 years in duration	No pain in controls, and ranging from 5 to 6.7 on VAS for patients with constant or intermittent pain	rsfcMRI revealed pain levels and emotional functional connectivity (striatum network) was positively correlated for intermittent pain but negatively correlated for constant pain
Poply et al. 2023 (95)	lumbar neuropathic pain caused by FBSS	T8 and T9	Tonic 4 weeks 40 Hz 4 weeks 4,000 Hz or 10,000 Hz in a randomized crossover design	Significant improvement in NRS (*p* = 0.001) at all frequencies compared to baseline	PET revealed SCS reduced metabolic activity by 50% or more in the thalamus, prefrontal cortex, insula, anterior cingulate cortex, and periaqueductal gray at 40 Hz and 4,000 Hz, but not at 10,000 Hz
Stancák et al. 2008 (107)	FBSS	T9–T11	3–4 days of SCS, SCS optimized to individual ranging from 45 to 85 Hz 36 s of SCS alone, heat pain to leg alone, or SCS + heat pain	VAS pain levels reported severe pain at 8.5 ± 0.8 (mean ± SD) before implantation and reduced pain levels at 5.25 ± 0.8 five days after implantation	fMRI revealed activation in primary sensorimotor cortex, posterior insula, and secondary somatosensory cortex during SCS There was decreased activation in primary motor cortices and primary somatosensory cortex during SCS Inferior temporal cortex and cerebellar cortex had significantly greater activation with combined SCS + heat pain compared to separate SCS and heat pain
Vetkas et al. 2025 (91)	PSPS or neuropathic pain	T8–T10	>1.5 years SCS, optimized per patient ranging from 40 to 1,200 Hz 6 min scan with 30 s SCS on/off cycles and 6 min off resting state between scans including 40 Hz and 1,200 Hz SCS conditions	Compared to preoperative values, SCS reduced NRS scores by 3, PCS by 10.5, and NPSI by 18.9	fMRI revealed optimal pain relief SCS was associated with reduced BOLD signals in the anterior cingulate cortex, midcingulate cortex, insula, supplementary motor area, sensorimotor operculum, parahippocampal gyrus, cerebellum, and left thalamus compared to SCS off Optimal stimulation increased BOLD activity in the periaqueductal gray and rostral brainstem compared to SCS off

All studies included in Table 1 were extracted through a systematic PubMed search with the search criteria “spinal cord stimulation” AND “chronic pain” AND “brain” AND “imaging” or found within references of relevant articles within this review and verified individually for inclusion by the study team. Studies were only included if medications were controlled or consistent throughout the study period. For short-term studies lasting less than 24 h, consistent medication throughout the study was presumed if not explicitly stated. Studies with *n* < 5 were excluded.

BOLD, blood oxygenation level dependent; CRPS, complex regional pain syndrome; EEG, electroencephalogram; FBSS, failed back surgery syndrome; FGD-PET, fluorodeoxyglucose positron emission tomography; fMRI, functional magnetic resonance imaging; NPSI, neuropahtic pain symptom inventory; NRS, numeric rating scale; PCS, pain catastrophizing scale; PET, positron emission tomography; PSPS, persistent spinal pain syndrome; PVAQ, pain vigilance and awareness questionnaire; rsfcMRI, resting state functional connectivity magnetic resonance imaging; SCS, spinal cord stimulation; T, thoracic; VAS, visual analogue scale.

Current literature has identified SCS-dependent metabolic, connectivity, and activity changes within nociceptive and affective-cognitive networks. A clinical trial used positron emission tomography (PET) scans to investigate SCS-induced changes within 57 individuals with intractable lumbar neuropathic pain (95). There were significant reductions in brain metabolic activity (59.5% thalamus; 52% prefrontal cortex, anterior cingulate cortex, and periaqueductal gray; 50% insular cortex; and 49% secondary somatosensory cortex) after 40 Hz and 4,000 Hz tonic SCS compared to baseline levels (95). Another PET study found that SCS significantly reduced pain and increased blood flow in the thalamus, anterior cingulate cortex, and prefrontal cortical areas (96). While the reduction of metabolic activity and increase in blood flow within the thalamus, anterior cingulate cortex, and prefrontal cortices seem contradictory, they can be explained by differences in stimulation protocols, the involvement of these areas in multiple networks, or differing activity across subregions. When SCS relieves pain, pathways within the anterior cingulate cortex may be simultaneously reduced and activated based on their specific involvement in affective pain processing, the attentional components associated with registering pain, or the emotional aspects of pain (96, 97). For example, during distraction from pain, the cognitive division of the anterior cingulate cortex exhibits a reduction of activity (43, 52), but the affective division of the anterior cingulate cortex shows increased activity (43). These regions are positioned within nociceptive and affective-cognitive networks, supporting the notion that SCS impacts both pain and cognitive processing. This theory is further corroborated by EEG and PET showing the alleviation of neuropathic pain coinciding with the normalization of metabolic activities within supraspinal structures responsible for pain perception, emotional/behavioral processing, and autonomic responses, including the anterior cingulate cortex, thalamus, orbitofrontal cortex, and primary somatosensory cortex (98). Comparing clinically optimal SCS settings to the SCS-off state in patients with persistent spinal pain syndrome or neuropathic pain revealed reduced BOLD fMRI signals in the anterior cingulate cortex, left insula, left supplementary motor area, and left sensory opercular cortex and increased BOLD activity in the periaqueductal gray and rostral brainstem (91). In contrast, findings in rats have highlighted significant increases in BOLD fMRI signals in areas known for processing the cognitive-emotional aspects (anterior cingulate cortex, amygdala, and insular cortex), location, and intensity of pain (primary somatosensory cortex and premotor cortex) following burst or tonic SCS (99). These findings indicate the multi-regional metabolic and structural effects of SCS in patients.

When comparing SCS on and off settings, a fMRI study found differences in resting state brain connectivity in sensory-cortical regions related to pain perception, including the insular cortex, primary and secondary somatosensory cortices, medial prefrontal cortex, and posterior cingulate cortex (100). Chronic pain may shift the representation of pain from sensory structures to limbic (emotional) regions, but these results indicate that SCS can shift limbic processing back toward sensory structures and higher-order cognitive processing. Specifically, in chronic pain, hyperactivity of the amygdala during pain generates feedforward inhibition, decreasing activity within the medial prefrontal cortex (30), coupling activity between the medial prefrontal cortex and thalamus (16), and impairing top-down cortical control mechanisms. In contrast, following SCS, there is a significant decrease in connectivity between the somatosensory cortex and limbic areas (e.g., amygdala), as well as increased connectivity between the somatosensory cortex and the default mode network (e.g., medial prefrontal cortex, posterior cingulate cortex) (100). It is possible that the pain relief from SCS may reduce the emotional processing associated with pain, allowing the somatosensory areas to be more involved in the default processing of pain. This connectivity shift following SCS may be a mechanism that improves overall top-down cortical control during pain processing. A few other key areas may serve as potential biomarkers for pain relief from SCS. Following SCS, there is deactivation of the bilateral medial thalamus and its connections to the rostral and caudal cingulate cortex and insular cortex (101). Ueno et al. found that differences in functional connectivity between middle anterior cingulate cortex and posterior cingulate cortex/precuenus could predict an individual's response to SCS (i.e., significant pain reduction or no significant pain reduction) with accuracy better than chance (102). With further investigation, this biomarker could potentially be used to estimate SCS responsiveness before surgical implantation, limiting unsuccessful surgeries. Importantly, it has been shown that opioid treatment and anesthetic blocks relate to increased activity in the anterior cingulate cortex (101, 103–106), so this must be taken under consideration when analyzing the functional connectivity of the anterior cingulate cortex. However, pain relief has been significantly correlated with both short-term deactivation in the anterior cingulate cortex and long-term deactivation in the thalamus (101), so the deactivation of the thalamus may be an additional biomarker for a patient's responsiveness to SCS treatment independent of medication status. Pain relief from SCS has also been negatively correlated with activity within the inferior olivary nucleus and the cerebellum (101). Interestingly, when simultaneous heat pain and SCS were applied to patients with neuropathic pain, there was significantly greater activation in the temporal and cerebellar cortices than when heat pain or SCS were applied separately (107), emphasizing these areas as additional overlapping regions within the pain and SCS networks.

The current EEG literature contrasts wave patterns observed in chronic pain between resting state and after SCS. Patients with chronic pain have lower alpha peak frequency than healthy controls (35–38, 108); however, studies have shown an increase in alpha peak frequency following SCS (86, 108). Patients with fibromyalgia show increases in theta, beta, and gamma power (35), but SCS decreases theta power (86, 108). Additionally, there is increased alpha power following SCS (86, 108). Shifts in alpha frequency have been shown to correlate with pain duration and intensity (13, 36, 38, 98). Importantly, these amplitudes were compared across healthy controls as well as patients with severe neuropathic pain syndrome at baseline and three months after implantation with SCS (98). The first marker of high-frequency SCS-induced pain relief is proposed to be the positive correlation between the alpha/theta peak power ratio in the frontal and primary sensory cortices and the self-reported improvements of pain (Oswestry Disability Index) (108). Interestingly, EEG revealed increased activity in the alpha1 frequency band within the dorsal anterior cingulate cortex with burst stimulation compared to both tonic stimulation and baseline activity (13, 87). There were differences in synchronized alpha1 frequency activity in the left and right cingulate cortex, and synchronized alpha1, beta2, and beta3 activity within the dorsolateral prefrontal cortex between tonic and burst stimulation (13, 87). There was also a decrease in gamma activity within the hippocampus after burst stimulation (13). Note that increased gamma was seen in patients with chronic pain (35). Both burst and tonic stimulation activate the pregenual anterior cingulate cortex, inferior secondary somatosensory cortex, posterior cingulate cortex, and the parahippocampus (13). Additionally, burst stimulation may also provide direct modulation of the spinothalamic pathways via the medial pain pathway (dorsal anterior cingulate cortex projections to the insular cortex) (13). In contrast to these EEG findings, MEG studies showed no significant differences between spectral features (i.e., alpha power ratio, and average power in theta, alpha, beta, and low-gamma frequency bands) between SCS-responsive and SCS-non-responsive individuals (109). However, there were significant increases in MEG sensory signal power below 3 Hz following burst stimulation when compared with tonic and sham SCS. The inconsistencies in MEG and EEG results may be due to inherent differences in the techniques themselves, as MEG is limited to tangential source orientation and EEG is not (110). Additionally, medication status may vary across patients and studies. Nevertheless, these studies find SCS-dependent changes spanning multiple spectral bands and key areas within both cognitive and pain networks. It is also possible that the activity changes resulting from SCS lead to large-scale structural changes in the brain over time, including within the precentral gyrus, precuneus, hippocampus, cerebellar posterior lobe, inferior temporal gyrus, inferior frontal gyrus, and middle frontal gyrus (111, 112). Specifically, SCS induced an increase in gray matter volume and a decrease in white matter volume within the premotor cortex/middle frontal gyrus volume, with the white matter changes correlating to pain relief (112). As patients with chronic migraine had significantly reduced cortical thickness (i.e., gray matter volume) in the middle frontal gyrus compared to healthy controls (40), SCS may be capable of normalizing these volumetric changes over time.

In sum:

• SCS decreases aberrant pain-related connectivity between the somatosensory cortex and limbic areas and increases connectivity between the somatosensory cortex and the default mode network (medial prefrontal cortex, cingulate cortex).
• The connectivity shift following SCS may reduce emotional processing and improve top-down cortical control during pain.
• SCS also reverses pain-related activity in cortical EEG (increase in alpha frequency and decrease in theta and gamma activity), especially in regions like the anterior cingulate cortex and dorsolateral prefrontal cortex.
• More controlled studies are needed to compare SCS effects on cognition across neurological conditions and to minimize confounding factors like medication use.

## Effects of SCS on cognition

Current research suggests SCS impacts language and speech processing, comprehension, memory, emotional functioning, conflict avoidance, and attention (82, 113–116). SCS has been shown to significantly improve attention in patients with varying neurological diseases (114). However, a patient's attention to their pain symptoms may influence the effectiveness of SCS treatment. One study investigated the mechanisms of attention in patients with chronic pain while they were receiving either burst, tonic, or sham SCS for two weeks (116). Participants received transcutaneous electrical pulses at the tibial nerve to induce somatosensory evoked potentials both while attending and not attending to the pulse. Importantly, medication dosages were kept consistent across SCS conditions. EEG was used to determine P300 amplitudes [event-related potential often used to measure attention (117)] and brain activation. Following the two-week stimulation period, there were reduced responses to the somatosensory evoked potential. When patients were not attending to the electrical pulse during tonic SCS, there was a decreased P300 amplitude and reduced activity in the somatosensory and motor cortices. Burst SCS, compared to sham SCS, decreased activity in the midcingulate cortex, insular cortex, supplementary motor area, and somatosensory cortices (116). Reduced activity in the supplementary motor area and somatosensory cortices suggests decreased activation of the dorsal attentional network that aids selective attention and stimulus responses. When an individual is not attending to a painful stimulus, there is decreased activity within the somatosensory cortices (116). SCS replicated this activity reduction (116). These findings suggest that SCS may induce similar changes in the somatosensory cortex as diverting a patient's attention away from pain. This is a potentially beneficial result of SCS, as attention to pain increases the intensity of the pain (52). This decrease in activity within the somatosensory cortex is in direct contrast with the over-activation (beta and gamma) seen in patients with chronic pain (35), identifying it as a potential neuromodulation target for SCS treatment or neuronal biomarker for attention in patients with chronic pain (Figure 1B).

Specific SCS parameters may also influence the executive and motivational functions critical for impulse control and responses to conflicting stimuli. In patients with Parkinson's disease, high cervical burst SCS was more effective than tonic stimulation at reducing pain and improving action cancellation, as measured by stop-signal reaction time (SSRT); however, these patients were also treated by levodopa (82). Further improvements in the cognitive-motivational aspects of pain were found with burst SCS in 38 rats (115). Rats showed significantly lower conflict-avoidance latencies compared to tonic SCS. These results suggest SCS may ameliorate the increased impulsivity and emotional decision-making seen in patients with chronic pain (31). However, given the complexity of chronic pain networks, further studies are needed to identify direct biomarkers of inhibited impulsivity. The somatosensory cortex is critical for processing painful stimuli (118) and distinguishing between painful and non-painful stimuli (16). It also functions in the identification of the emotional significance of a stimulus, generation of emotional states, regulation of emotion (119), and empathy (120). Structural and functional changes in the somatosensory cortex have been associated with abnormal emotional regulation (5). Given that SCS modulates connectivity between the somatosensory cortex and the limbic system, SCS may modulate emotional regulation through somatosensory cortical connectivity. Additionally, the functional connectivity patterns of the striatal network (caudate nucleus, putamen, and globus pallidus), important in emotion and reward circuitry, are significantly altered in patients with pain and SCS systems when compared to healthy controls (121–123). Pahapill and colleagues have further discovered that striatum network indexes of patients with constant neuropathic pain were directly correlated with their duration of pain relief with SCS therapy (122), suggesting normalization of the striatal network over time. Interestingly, pain levels and emotional functional connectivity were positively correlated for intermittent pain but negatively correlated for constant pain (121). Functional changes in pain and emotion following short-term SCS have also been correlated to the middle occipital gyrus, inferior parietal gyrus, middle temporal gyrus, supramarginal gyrus, rolandic operculum, and precuneus (124). Likely, emotional functioning is affected by multi-network stimulation during SCS in addition to the emotional processing involved with the relief of long-term pain. One study suggests that SCS influences pain perception and the emotional aspects of pain concurrently; specifically, SCS modulated neuronal activity in regions associated with pain modulation and emotional regulation, including the anterior cingulate cortex, thalamus, and prefrontal cortex, and led to effective reduction of neuropathic pain (96) (Figure 1C). A large multi-center study compared cognitive performance in 269 patients at six and twelve months following SCS implantation (113). Patients showed significant improvements in mental and emotional functioning, specifically for catastrophizing (PCS), and quality of life (EQ-5D) (113). After a year of using SCS, over 80% of individuals were satisfied with their therapy, 89.3% reduced their pain medication, and 19% ceased pain medication altogether (113). Interestingly, after 3 months of high-frequency SCS, there is a correlation between the minimum clinically important difference value of the Pittsburgh sleep quality index and the increased strength in connectivity between dorsolateral prefrontal cortex and anterior insular cortex (125). While it is possible that some of these cognitive, emotional, and lifestyle improvements could be attributed to decreased medication, the study showed that clinically significant improvement on the PHQ-9 and STA scales for depression and anxiety was directly associated with greater pain relief (113). Other studies suggest that opioid use in the treatment of chronic pain is not significantly associated with cognitive impairment (54, 55, 57). It is difficult to disentangle whether the changes in cognition and emotional functioning in patients with chronic pain are due to the decreased perception of pain following SCS treatment or the stimulation itself. However, system-wide improvements resulting from SCS are clearly evidenced.

Although memory and working memory deficits are main cognitive complaints of patients with chronic pain, there are very few studies linking SCS treatment to memory improvements in these patients. One study showed that SCS significantly improved performance on the Wechsler Memory Scale of patients with various neurological disorders (114). However, chronic pain was not the main criterion for subject inclusion; instead, this study group consisted of patients with cerebral palsy, dystonia, muscular sclerosis, and torticollis. It is possible that memory may be affected by the changing plasticity within limbic regions of the amygdala and hippocampus that are integral to learning and memory functions (33, 45, 47–49, 53) (Figure 1D). While the progression from acute to chronic and persistent pain can instigate activity-induced plasticity in the limbic-cortical circuitry (30), following SCS there is a significant decrease in connectivity between the somatosensory cortex and the limbic areas (e.g., amygdala), as well as increased connectivity between the somatosensory cortex and the default mode network (e.g., medial prefrontal cortex, posterior cingulate cortex) (100). SCS may impact memory through changes in the limbic network, specifically in amygdala connectivity. However, further studies are needed to investigate how the supraspinal changes resulting from SCS may directly affect memory in patients with chronic pain.

In sum:

• SCS influences cognitive functions, including attention, language, memory, and emotional regulation.
• EEG studies show that SCS reduces activity in the somatosensory cortex in patients with chronic pain, similar to when attention is diverted from pain.
• Burst SCS improves executive control and emotional processing. These effects are linked to changes in somatosensory-limbic connectivity and the normalization of the striatal network.
• Few studies have directly linked SCS to memory improvement in chronic pain. More targeted studies are needed to confirm its effects on memory in chronic pain populations.

## Discussion

SCS can improve pain perception and related physical well-being, yet the mechanisms by which SCS affects cognition remain largely unknown. This review compares the current literature on the supraspinal changes involved with pain processing and cognition in chronic pain patients to the supraspinal and cognitive changes associated with SCS. There are various regions where SCS may modulate both pain processing and other cognitive processes, including attention, executive and emotional functioning, and memory (Figure 1). Specifically, we highlight the anterior cingulate cortex, amygdala, thalamus, and somatosensory cortex as overlapping regions for pain processing, cognition, and SCS treatment. Uncovering overlapping biomarkers for pain and cognition could potentially aid in individualizing SCS and further enhancing adaptive SCS systems. Recently, fMRI-based biomarkers have been suggested for optimization of pain treatment with SCS (91, 102), either by using prognostic connectivity measurements between the anterior cingulate cortex and precuneus to predict potential for pain relief (102) or by using unique BOLD activation patterns associated with optimal stimulation patterns (91). It is critical for future research to investigate whether the reduction of pain following SCS treatment correlates with cognitive effects in order to better individualize pain treatment and induce favorable cognitive shifts.

Future studies are also needed to disentangle the cognitive effects of SCS from the nuanced changes associated with variations in stimulation settings, location, habituation, spread, and medication dosage. While positive cognitive effects of SCS have been seen within a cohort of individuals with varying neurological disorders (114), SCS can have dissociable effects on patients with spinal cord injury, gait or postural impairments, or chronic pain. Further, a systematic review found inconclusive results when they investigated cognition across transcutaneous stimulation and various other spinal cord injury interventions (126). One fundamental limitation of determining the cognitive effects of SCS in pain research is that the supraspinal activation and activity changes following SCS may stem partly from the reduction of medications and their associated side effects (127, 128). However, other evidence found no significant link between cognitive impairments and opioid use in chronic pain treatment, implying the cognitive changes following SCS may be due to decreased perceived pain rather than a reduction of medication (54, 55, 57, 113). Overall, the current literature indicates that SCS enhances cognitive, attentional, and emotional functioning along with pain relief, though outcomes may vary by individual, location, and medication status. Future studies may investigate these variations for placebo effects (inclusion of sham stimulation), correlation of reduced SCS therapy due to adaptation or habituation, ineffective current spread due to lead migration and scar formation, and thoracic/abdominal paresthesia. Additionally, further studies are needed to parse how successful and unsuccessful pain management with SCS can influence quality of life, cognition, emotion, memory, and attention in patients with chronic pain. Pain management and cognitive effects following SCS may also vary based on stimulation type and frequency. Some studies show that burst SCS, compared to tonic SCS, yielded more significant improvements in conflict-avoidance and the cognitive-motivational aspects of pain (13, 82, 84, 85, 115). Additionally, there are differences in synchronized alpha1 frequency activity in the cingulate cortex, and synchronized alpha1, beta2, and beta3 activity within the dorsolateral prefrontal cortex between tonic and burst stimulation (13, 87). However, how SCS settings affect cognitive processes like attention, memory, and other areas of executive and emotional functioning is understudied. Elucidating the mechanisms behind these cognitive effects is a critical step to optimizing SCS.

SCS remains a valuable, minimally invasive treatment option for drug-refractory chronic pain that can improve pain perception and related physical well-being. Our comprehensive review highlights the anterior cingulate cortex, amygdala, thalamus, and somatosensory cortex as regions where SCS may modulate both pain processing and various other cognitive processes, including attention, executive and emotional functioning, and memory. Recognizing activity changes within these key supraspinal regions during SCS treatment may help identify biomarkers for pain treatment, induce favorable cognitive shifts, and inform cortical feedback regions for adaptive SCS systems. Further studies are necessary to disentangle the supraspinal effects stemming from the decreased perception of pain following SCS treatment, the reduction of medication coinciding with the SCS management of pain, and the stimulation itself.
